# Immunoglobulin-G4 Related Disease: A Rare Entity with Many Clinical Faces – Literature Review and Case Illustration

**DOI:** 10.31138/mjr.150825.era

**Published:** 2026-03-01

**Authors:** Vijay Krishnan R, Hariharan Seshadri, Suganya Balachandran, V Aribalaji, Umadevi T B

**Affiliations:** Institute of Internal Medicine, Madras Medical College and Rajiv Gandhi Government General Hospital, Chennai, Tamil Nadu, India

**Keywords:** immunoglobulin G4-Related disease, autoimmune pancreatitis, orbital pseudotumor, venous thrombosis, immunoglobulin G4

## Abstract

**Introduction:**

Immunoglobulin-G4 Related Disease (IgG4-RD) is a group of multi-system, fibro-inflammatory conditions characterised by elevated IgG4 levels and unique histopathological features. Clinical presentations of the disease are highly variable, albeit there exist distinct phenotypes in the presentations of the disease. IgG4-RD is a differential diagnosis that should be missed in any case of chronic inflammatory and tumefactive pathology.

**Case Discussion:**

We present the case of a 47-year-old diabetic female who presented with features suggestive of acute pyelonephritis. On further evaluation, our findings unmasked a rare constellation of pancreatitis, orbital pseudotumor, and venous thrombosis. After an astute clinical observation by our team, we were able to decipher these as features of distinct phenotypes under the IgG4-RD spectrum with minimal overlap (pancreato-hepatobiliary disease, head and neck disease, and retroperitoneal disease groups, respectively).

**Literature Review:**

We have presented an extensive review of the recent literature pertaining to the etiopathogenesis, clinical features, diagnostic protocol and management consensus for IgG4-related disease. A detailed account of the possible clinical presentations and work-up strategy for suspected patients has been emphasised in particular.

**Conclusion:**

IgG4-RD is diverse clinico-pathological entity that warrants a high degree of clinical suspicion. Once identified, efforts must be made to screen all possible foci of disease activity characteristic to the disease, as a multi-focal disease presentation of IgG4-RD is not unlikely.

## INTRODUCTION

Immunoglobulin-G4 Related Disease (IgG4-RD) is a constellation of diverse fibro-inflammatory disease entities presenting with elevated IgG4 levels, characteristic histopathological findings, and multi-system involvement. The clinical presentations of the disease vary greatly depending on the organs involved. Often a disease of the middle-aged and elderly with male predisposition, it follows a chronic course and typically involves the pancreas, salivary and lacrimal glands, orbital adnexa, lymph nodes, and retroperitoneum. Often, the suspicion of IgG4-RD comes from an incidental finding in radiographic studies done for other conditions. When seen in conjunction with the chronic clinical history, these features point towards the diagnosis of the disease. We present one such case of a patient with acute pyelonephritis, where further evaluation revealed diffuse pancreatic enlargement, orbital pseudotumor, venous thrombosis, all of which are tell-tale features within the IgG4-related disease spectrum, but belong to distinct clinical phenotypes of the disease. We further provide a review of the current literature highlighting the heterogeneous nature of clinical manifestations in IgG4-RD and the need for clinicians to extensively screen for all possible foci of disease activity in all patients of IgG4-RD despite predominance of one specific clinical phenotype in the individual. This manuscript has been formulated in compliance with the CABARET Guidelines for Case-based reviews.^1^

## CASE DISCUSSION

A 47-year-old female presented to our hospital with chief complaints of high-grade intermittent fever with chills and rigors, non-bilious vomiting, and dull-aching abdominal pain for the past three days. The abdominal pain was more in the flanks with radiation to the groin, and associated with burning sensation during micturition. She has a history of type 2 diabetes mellitus and hypothyroidism for the past two years on regular treatment. She is a peri-menopausal home-maker from lower socio-economic status (according to modified Kuppusamy scale 2025).^2^ The patient had a history of abdominal discomfort (early satiety and bloating sensation after meals), significant unintentional weight loss (around 8–10 kg), easy fatigability, and bilateral knee pain for the past two months, and boring type, holocranial headaches for the past three weeks. On examination, the patient was febrile (temperature = 101.7^0^F), pale, thin-built (BMI = 18.7 kg/m^2^), and poorly nourished. Her vitals were – blood pressure: 100/60 mm Hg, pulse rate: 115/min, respiratory rate: 22/min, capillary blood glucose: 356 mg/dL. Bilateral renal angle tenderness was present. Cardiopulmonary and nervous system examinations were unremarkable. Results of the laboratory investigations are given in **Table 1***.* Urine culture revealed the growth of *Klebsiella pneumonia spp.* with 105 CFU/mL, sensitive to piperacillin-tazobactam, cefazolin, amikacin, and meropenam. Ultrasound study of abdomen and pelvis showed bilateral bulky kidneys with perinephric fat stranding – features suggestive of acute pyelonephritis. The patient was initially given empirical antibiotic therapy (intravenous ceftriaxone 1 g twice daily), later shifted to intravenous piperacillin-tazobactam 4.5 g thrice daily based on the antibiogram. Required electrolyte correction measures were administered.

**Table 1.** Baseline laboratory investigations of the patient.

**Parameter**	**Value**	**Parameter**	**Value**
Haemoglobin (g/dL)	**9.1**	Serum Sodium (mEq/L)	**128**
WBC count (per mm^3^)	**14,200**	Serum Potassium (mEq/L)	**3.2**
Platelet count (per mm^3^)	366,000	Fasting plasma glucose (mg/dL)	**174**
Serum Urea (mg/dL)	**79**	Postprandial plasma glucose (mg/dL)	**329**
Serum Creatinine (mg/dL)	**1.3**	Serum TSH (μIU/mL)	2.8
Total bilirubin (mg/dL)	0.9	Serum Free T4 (ng/dL)	1.4
Direct bilirubin (mg/dL)	0.5	Blood pH	7.4
SGOT/AST (IU/L)	27	Plasma pCO2 (mm Hg)	21.5
SGPT/ALT (IU/L)	13	Plasma bicarbonate (mmol/L)	**16.4**
ALP (IU/L)	**257**	C-Reactive protein (mg/L)	**34**
Serum Total protein (g/dL)	**5.7**	Urine acetone	Negative
Serum Albumin (g/dL)	**2.9**	Urine pus cells (per HPF)	**17**
Serum Amylase (IU/L)	82	Sputum Acid-fast bacilli	Negative
Serum Lipase (IU/L)	56	Sputum CBNAAT	Negative

Abnormal values have been given in bold for the ease of the readers.

WBC: white blood cell; SGOT: serum glutamic-oxaloacetic transaminase; AST: aspartate aminotransferase; SGPT: serum glutamic pyruvate transaminase; ALT: alanine aminotransferase; ALP: alkaline phosphatase; TSH: thyroid-stimulating hormone; HPF: high-power field; CBNAAT: cartridge-based nucleic acid amplification test (for Mycobacterium tuberculosis).

Contrast-enhanced computed tomography (CECT) scan of abdomen was done to look for complications of pyelonephritis and it revealed further details – bilateral bulky kidneys with perinephric fat stranding; thrombosis of segmental renal veins bilaterally; partial IVC thrombosis extending from intra-hepatic to supra-renal portion; diffuse enlargement of pancreas with loss of pancreatic cleft definition showing minimal peri-pancreatic fat stranding with a sausage-shaped appearance; presence of para-aortic and mesenteric lymphadenopathy; no evidence of retroperitoneal or large vessel pathology. Computed tomography of chest showed diffuse non-homogenous areas of altered attenuation with mild bronchial wall thickening and few pleuro-parenchymal bands – features suggestive of small airway disease. Electrocardiography and echocardiography studies were unremarkable.

In light of the above findings, the possibilities of an underlying autoimmune association (due to multiple organ involvement), acquired thrombophilia (secondary to sepsis, causing a pro-thrombotic state), or malignancy (due to clinical signs and lymphadenopathy) were considered. The autoimmune workup returned negative for anti-nuclear antibodies, anti-dsDNA, β_2_-glycoprotein (IgG and IgM), lupus anticoagulant, anti-Ro/La, and anti-cardiolipin (IgG and IgM) antibodies. Evaluation for occult malignancies was unremarkable with normal oesophago-gastro-duodenoscopy study, negative stool occult blood, and normal levels of serum carcinoembryonic antigen (CEA) of 2.4 ng/mL (normal range: < 5 ng/mL). Coagulation profile revealed the PT, INR, aPTT, serum fibrinogen values to be 12.6 seconds, 1.08, 37.4 seconds, and 274 mg/dL, respectively, ruling out the possibility of septic/thrombophilic complications.

During the course of evaluation, the patient’s headache had aggravated. Clinical examination did not reveal any focal neurological deficits. Ocular fundus examination showed no signs of papilledema and computed tomography of brain was unremarkable. Cerebrospinal fluid (CSF) analysis was within normal limits. Magnetic resonance imaging (MRI) of brain was then done, which revealed a T1/T2-weighted hypo-intense soft tissue thickening in peri-optic space showing no diffusion-restriction, extending into orbital apex – possibility of orbital pseudotumor. The headache was managed symptomatically after ruling out imminent aetiologies. Connecting the dots between middle age group, suba-cute onset of weight loss and early satiety, presence of sausage-shaped diffuse pancreatic enlargement, orbital pseudotumor, and venous thrombosis, our team suspected the possibility of IgG4-related disease (IgG4-RD). Serum IgG4 level was then measured and found to be 240 mg/dL (cut-off for diagnosis > 135 mg/dL).

**Figure 1.**
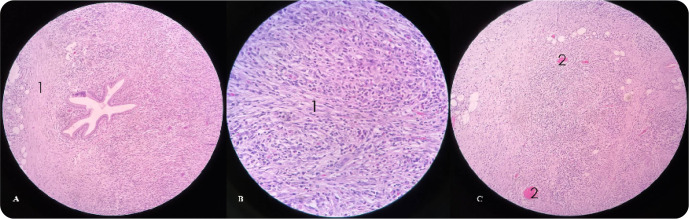
Histopathological study of the pancreatic biopsy specimen (with haematoxylin-eosin staining); A & C: low-power images, B: high-power image. (1) Some pancreatic lobules exhibiting parenchymal atrophy, surrounded by storiform fibrosis, lymphocytes, plasma cells and eosinophilic infiltrates. (2) Foci exhibiting lymphoplasmacytic infiltrates within the lumen and vessel wall (obliterative phlebitis)

In order to obtain a definitive diagnosis, the patient was posted for pancreatic biopsy under imaging guidance after stabilisation and treatment of infection. Multiple sections from the pancreatic specimen showed lobules of pancreatic acini with some lobules exhibiting parenchymal atrophy, surrounded by stori-form fibrosis, lymphocytes, plasma cells, and eosinophilic infiltrates. The fibro-inflammation was centred around the ducts and lobules and was seen infiltrating into the adjoining fibro-fatty stroma. Few foci exhibited lympho-plasmacytic infiltrates within the lumen and vessel wall (obliterative phlebitis). Remnant clusters of islet cells were seen (**Figure 1**).

In accordance to the 2020 Revised Comprehensive Diagnostic Criteria (RCD) for IgG4-RD (refer **Table 3**), a definitive diagnosis of Immunoglobulin G4-Related Disease was made on the grounds of positive radiological (diffuse pancreatic enlargement, orbital pseudotumor), serological (serum IgG4 level > 135 mg/dL), and pathological evidence (lympho-plasmacytic infiltration, storiform fibrosis, obliterative phlebitis).

**Table 3.** The 2020 Revised Comprehensive Diagnostic (RCD) for IgG4-related disease.

**S. No.**	**Criteria**	**Our Patient**
**[1]**	**Clinical and Radiological features** One or more organs show diffuse or localised swelling or a mass or nodule characteristic of IgG4-RD. In single organ involvement, lymph node swelling is omitted.	Diffuse pancreatic enlargement, orbital pseudotumor, venous thrombosis present
**[2]**	**Serological Diagnosis** Serum IgG4 levels greater than 135 mg/dL	Serum IgG4 = 240 mg/dL
**[3]**	**Pathological diagnosis** Positivity for *two* of the following three criteria: (i) Dense lymphocyte and plasma cell infiltration with fibrosis	Lympho-plasmacytic infiltration, storiform fibrosis, obliterative phlebitis present
	(ii) Ratio of IgG4-positive plasma cells/IgG-positive cells greater than 40% and the number of IgG4-positive plasma cells greater than 10 per high powered field	
	(iii) Typical tissue fibrosis, particularly storiform fibrosis, or obliterative phlebitis	
**Diagnosis:** Definite: [1] + [2] + [3] Probable: [1] + [3] Possible: [1] + [2]		Definitive diagnosis made in our patient (Criteria [1]+[2]+[3] satisfied)

Adapted from Umehara H, et al. (2021), The 2020 revised comprehensive diagnostic (RCD) criteria for IgG4-RD.**^19^**

Screening was done in order to identify involvement of other organ systems in the IgG4-RD spectrum. Serum cortisol at 8 a.m. (6.3 μg%; normal range: 5.2 – 22.4 μg%) and serum prolactin (14 ng/mL; normal <25 ng/mL) levels were ascertained to look for hypophyseal involvement and were normal. The 24-hour urine protein level was 126 mg. Schirmer’s test was negative for ocular disease. High-frequency ultrasound study of parotid and submandibular regions showed no swelling of salivary glands.

The patient was thus started on oral prednisolone at 1 mg/kg/day for a duration of four weeks after ruling out possible contraindications. While the steroid dosage is higher than the standard 0.6 mg/kg/day, the local experience at our institution has shown that initiating patients on prednisolone at 1 mg/kg/day has a lower rate of relapse in comparison to the standard dosage in the Indian population.

The antibiotic therapy for acute pyelonephritis was continued for two weeks. She was started on intravenous heparin 5000 IU four times a day for the venous thrombosis with close monitoring of coagulation parameters. Later, she was shifted to oral Apixaban 10 mg twice daily in the view of better renal safety profile. Ultrasound monitoring was done to monitor the thrombus size and progressive vascular recanalisation. Anti-diabetic medications were reviewed to ensure strict glycaemic control. After the resolution of the acute infection, the patient was discharged with oral prednisolone and oral Apixaban 5 mg twice daily, and was asked to review after four weeks.

**Figure 2.**
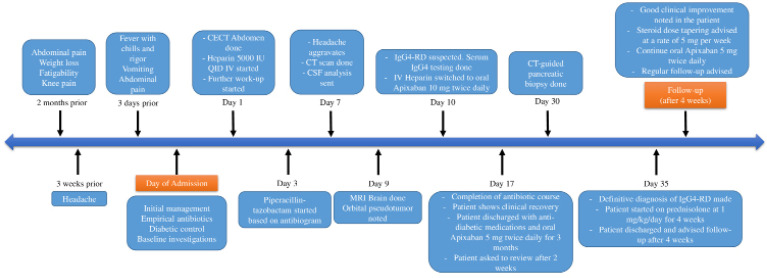
Clinical timeline of the reported case. IgG4-RD: IgG4-related disease; CECT: Contrast enhanced computed tomography; QID: Four times daily (in Latin); CT: Computed tomography; CSF: Cerebrospinal fluid; MRI: Magnetic resonance imaging.

During the follow-up visit, there was a definitive improvement in appetite, with a reduction in severity of bloating, fullness, and early satiety. Repeat imaging studies showed reduction in pancreatic size and partial recanalisation of thrombosed vessels. She was advised to gradually taper the steroid dose by 5 mg every successive week. The patient is currently on oral prednisolone 10 mg/day and oral Apixaban 5mg twice daily, and has been advised to be on regular follow-up. The timeline of the patient’s clinical course is depicted in **Figure 2**.

## METHODOLOGY

### Search Strategy

A comprehensive literature review was conducted through extensive searching of PubMed, Scopus, Web of Science, and Google Scholar databases. Medical Subject Headings (MeSH) and relevant keywords were used to construct the search strategy for the retrieval of relevant literature. Key terms included - “Immunoglobulin G4-Related Disease”, “IgG4-RD”, “Immunoglobulin G4”, “IgG4”, “Autoimmune”, “Autoimmune pancreatitis”, “Orbital pseudotumor”, “Venous thrombosis”, “Mikulicz disease”, “Ormond’s disease”, “Pancreato-hepatobiliary”, “Head and neck disease”, “Retroperitoneal disease”, “Aortic disease”, “Large vessel disease”, “sclerosing cholangitis”, “Reidel’s thyroiditis”, “Pachymeningitis”, “Hypophysitis”, “Pseudotumor”, “Tumefaction”, “Pleuritis”, “Pneumonitis”, “Pericarditis”, “Periaortitis”, “Neuropathy”, “Nephritis”, “Lymphadenopathy”, “Lymphoplasmacytic infiltrates”, “Storiform fibrosis”, “Obliterative phlebitis”, “Sausage-shaped pancreas”, “Pathophysiology of IgG4-RD”, “Diagnosis of IgG4-RD”, “Steroid therapy”, “Prednisolone”, “Immunosuppressant therapy”, “Rituximab”, “Azathioprine”, “Mycophenolate mofetil”, “Calcineurin inhibitors”, “Relapse”. Controlled vocabulary was used in conjunction with appropriate Boolean operators to refine the search and ensure a thorough review of the pertinent literature.

### Inclusion Criteria

Studies providing insights on the pathophysiology, epidemiology, clinical manifestations, diagnostic work-up, therapeutic management, clinical prognosis, and future research directions of IgG4-RD were considered. Original articles, case reports, and review articles published in peer-reviewed journals were included. Publications were considered without any time, demographic or geographic restrictions to facilitate a comprehensive review. Only studies written in English language were included.

### Data Collection

An initial screening of article titles and abstracts was performed to identify the relevant works. Studies lacking alignment or sufficient clinical insights were excluded. The screened studies were subjected to an in-depth full-text review to assess their relevance, significance and contribution to the understanding of IgG4-RD. References from selected articles were screened to identify any additional relevant studies.

### Assessment Criteria

Formal risk-of bias assessment tools were not applied to the selected studies as this is a narrative literature review. However, the included studies were thoroughly scrutinised for scientific rigor, topic relevance, clinical insights, and novelty in findings, diagnostic approach, therapeutic strategies, and clinical outcomes.

## LITERATURE REVIEW

Immunoglobulin G4-related disease (IgG4-RD) is an immune-mediated, heterogeneous cluster of fibro-inflammatory conditions characterised by elevated levels of serum IgG4, lymphoplasmacytic infiltration, and formation of tumefactive lesions with characteristic histopathological features. While the involvement of nearly every organ has been reported in the literature, the most common organs involved are pancreas, salivary and lacrimal glands, retroperitoneum, kidneys, lungs, lymph node, and orbital adnexa.^3^

### Epidemiology and History

The epidemiology of IgG4-RD has been debatable. While cases have been reported worldwide, a majority of the reported cases are from Japan alone. Japanese studies report an annual incidence of 0.28 – 1.08/100,000 population and prevalence of 1 in 600,000 persons.^4^ The disease incidence typically peaks between the 5^th^ and 7th decades of life, although paediatric cases of IgG4-related dacryoadenitis have been reported.^5,6^ There is an overall male predilection (males:females = 1.6 – 4:1) for the disease, however disparities exist based on the site of involvement (thyroiditis, dacryoadenitis and sialadenitis are common in females).^5^ Indian studies have reported a male:female ratio of 1:0.94 and mean age of patients to be 41.4 years.^7^

Historically, the diseases under the IgG4-RD spectrum were considered as independent entities. In 2001, Hamano et al*.* described elevated serum IgG4 levels in cases of autoimmune pancreatitis, which eventually led to discovery of IgG4+ plasma cells in the inflammatory lesions.^8^ Kanisawa *2004* proposed the term ‘IgG4-related autoimmune disease’ to refer to a cluster of diseases with similar findings.^9^ An International Symposium on IgG4-Related Disease (Boston, 2011) finally approved the term ‘IgG4-related disease’ over the other alternative names proposed for this disease spectrum.^10^

### Pathogenesis

The pathogenesis of IgG4-RD has not been clearly understood and is thought to be associated with excessive IgG4 antibody production. IgG4 is the least abundant among the IgG subclasses (constituting < 5% of total IgG). The inability of IgG4 to form effective immune complexes or activate the complement pathway made it less likely to be implicated to participate in any autoimmune aetiology. However, the demonstration of IgG4 antibodies against carbonic anhydrase II and lactoferrin in cases of type 1 autoimmune pancreatitis has challenged the earlier hypothesis.^11^ Our current understanding is centred around autoreactive B-cells and T-cells. The predominant Th2 response seen in IgG4-RD is due to the over-production of cytokines such as IL-4, IL-10, IL-13, and TGF-β by T-regulatory cells.^12^ This increases IgG4 and IgE production, interferes with Ig-class switching, and promotes a fibrotic phenotype in the affected individuals.

Both genetic and environmental triggers seem to play a role in the pathogenesis. Several HLA (HLADRB1*04:05, HLA-DQB1*04:01, HLA-DRB1*03) and non-HLA (P2RX3, TOP1, PRSS1, SPINK1) gene associations with IgG4-RD have been described in the literature.^13^ Environmental exposures to industrial solvents, metal dust, asbestos, and history of smoking and atopy (allergic rhinitis, asthma, atopic dermatitis) have been shown to trigger IgG4-RD spectrum diseases.^14^ Association with allergic diseases is seen in 40% of the patients (due to Th2 phenotype), more commonly with head and neck involvement, and these are non-seasonal and damaging in nature.^11^ A positive family history of other autoimmune diseases was associated with an earlier age of onset of IgG4-RD and an unfavourable disease prognosis.^15^

### Clinical Presentation

The clinical manifestations of IgG4-RD are quite variable and subtle. It is often an incidental finding on radiographic or histopathological evaluation done for other aetiologies. It often presents with tumefactive lesions in one or more organs with or without constitutional symptoms (fever, fatigue, and weight loss). Lymphadenopathy is a common finding that often occurs in conjunction with other elements of the disease, albeit it could also be the only finding. Although mostly asymptomatic, symptomatic lymphadenopathy is seen due to mass effects on surrounding structures. Mediastinal, hilar, intra-abdominal, and axillary lymph nodes are most commonly involved. Diagnosis solely based on lymphadenopathy can be challenging due to non-specific histological findings. Infections, lymphoma, sarcoidosis, multicentric Castleman disease are possible differentials for IgG4-related lymphadenopathy.^14^

IgG4-RD spectrum encompasses a wide-range of presentations depending on the organ(s) involved. Type 1 autoimmune pancreatitis (AIP) and Mikulicz disease are the most common disease manifestations.^11^ Other noteworthy presentations include pulmonary (inflammatory pseudotumor, pleuritis, interstitial pneumonitis, lymphomatoid granulomatosis), renal (tubulo-interstitial nephritis with low C3 and C4 levels), skin (cutaneous pseudolymphoma, hyperplasia with eosinophilia, angiolymphoid hyperplasia with eosinophilia), breast (sclerosing mastitis, inflammatory pseudotumors), pericarditis, periaortitis, periarteritis, neuropathy, and prostatitis.

Wallace et al. identified four broad domains of presentations of the disease – pancreato-hepato-biliary disease, retroperitoneum and aortic disease, head and neck limited disease, and Mikulicz and systemic disease.^16^ **Table 2** discusses the manifestations, clinical features and differential diagnoses for these subgroups. Some characteristics of these subgroups deserve a mention – retroperitoneal/aortic disease is characterised by more fibrosis, less inflammation, and near-normal IgG4 levels, while patients with Mikulicz disease display very high IgG4 levels; head and neck diseases have a predilection for female sex, Asian race, and atopy.

**Table 2.** Clinical phenotypes of IgG4-related disease.

**Disease Pattern**	**Pancreato-hepatobiliary disease**	**Retroperitoneum and aortic disease**	**Head and neck limited disease**	**Mikulicz and Systemic disease**
**Manifestations**	• Autoimmune pancreatitis type 1 • Sclerosing cholangitis	• Retroperitoneal fibrosis (Ormond’s disease) • Aortitis and Large vessel disease Rarely, • Thyroiditis • Sclerosing mesenteritis • Mediastinitis	• Chronic sclerosing sialadenitis (Kuttner’s tumour) • Dacryoadenitis • Orbital adnexa involvement (orbital myositis, orbital pseudotumour) • Pachymeningitis • Thyroiditis (Riedel’s, fibrous variant of Hashimoto’s thyroiditis) • Hypophysitis	• Classical Mikulicz disease • Chest/abdomen manifestations of IgG4-RD
**Clinical features**	• Painless obstructive jaundice • Epigastric mass • Endocrine and exocrine pancreatic insufficiency • Profound weight loss, fatigue, arthralgia • Elevation in hepatic and pancreatic enzyme levels	• Back/flank/groin pain (dull-aching, ill-localized, better response to NSAIDs than opioids) • Ureteral obstruction • Hydronephrosis • Renal failure • Thrombophlebitis/deep vein thrombosis (due to inferior vena caval obstruction) • Vascular dissection and aneurysm	**Salivary/Lacrimal gland disease** Enlargement of salivary (commonly submandibular) and lacrimal glands **Orbital disease** Painless periorbital swelling, proptosis, and visual disturbances **Meningeal disease** Headaches, mass effects, and focal neurological deficits **Thyroid disease** Hard, enlarged and adherent thyroid gland with obstructive symptoms (dyspnoea, dysphagia, stridor). Parathyroid involvement may be seen **Pituitary disease** Loss of weight, appetite and libido, diabetes insipidus, headache, visual field defects	Bilateral, painless, symmetric enlargement of lacrimal and salivary glands without signs of dryness (dry eyes, dry mouth) and arthralgia
**Differential Diagnosis**	Pancreatic neoplasm, cholangiocarcinoma, autoimmune pancreatitis type 2, primary sclerosing cholangitis	**Retroperitoneal disease** Lymphoma, sarcoma, infections, drugs, radiotherapy, Erdheim–Chester disease **Vascular disease** Giant cell arteritis, Takayasu arteritis (both have a predilection for primary aortic branches, but are spared in IgG4-RD), sarcoidosis, histiocytosis, lymphoma	**Autoimmune** – Sjögren’s syndrome, systemic sclerosis, sarcoidosis, granulomatosis with polyangiitis (formerly Wegener’s), eosinophilic granulomatosis (Churg-Strauss syndrome) **Malignancy** – Lymphoma, sarcoma, Langerhans cell histiocytosis **Thyroid disease** – Graves’ disease, thyroid neoplasm/lymphoma, **Others** – Infections, allergy, drug-induced	Sjögren’s syndrome, lymphoma, sarcoidosis, lymphoepithelial sialadenitis, infections

Adapted from Wallace ZS, et al. (2019), Clinical phenotypes of IgG4-related disease: an analysis of two international cross-sectional cohorts.^16^

IgG4-RD follows an indolent clinical course with a variable prognosis. The disease may go into spontaneous remission, follow a remitting-relapsing course, or may persist and worsen with the onset of new complications. A good response to steroid therapy is a hallmark sign of IgG4-RD.^14^ Late identification of the disease results in advanced progression and organ failure due to local pressure effects, secondary sclerosis, and obstruction. Pancreatic insufficiency, biliary obstruction, liver cirrhosis, portal hypertension, ureteric obstruction, aortic dissection, and aneurysms are some common complications of the disease. IgG4-RD also furnishes an elevated risk of malignancies of the pancreato-biliary tree, lung, thyroid, prostate, stomach, and non-Hodgkin lymphoma.^14^ Symptom Severity Index (SSI) has been developed to assess the symptom burden in patients with IgG4-RD.^17^

### Investigation Modalities

A systematic approach to IgG4-RD requires a comprehensive workup involving radiological, histopathological and serological testing. Radiographic modalities demonstrate classical findings of the disease, and are used to identify organ involvement and monitor the disease activity. In patients with type 1 AIP, computed tomography (CT) images show a sausage-shaped pancreatic enlargement with loss of lobulation, irregular pancreatic duct, positive halo sign, and peri-pancreatic fat stranding.^14^ CT chest in IgG4-related lung disease shows solid nodular, broncho-vascular, alveolar interstitial patterns and ground glass opacities. CT images showing medial displacement of ureter is suggestive of compressive effects in retroperitoneal disease. 18-FDG positron emission tomography (18-FDG PET) is useful for monitoring the inflammatory activity of the disease. Patients with positive findings on PET scan have a better response to steroid therapy than those with negative findings at onset of treatment.^14^

Serological evaluation reveals elevated serum IgG4 levels in about 70% of cases. While a mild elevation (1.5 – 5 g/L) is non-specific, marked elevation in IgG4 levels (>5 g/L) has 90% specificity for the disease. A normal IgG4 level (<1.4 g/L) does not rule out the disease. Elevated erythrocyte sedimentation rate (ESR), C-reactive protein (CRP), anti-nuclear antibodies (ANA), and hypo-complementaemia are other possible serological findings in IgG4-RD patients.^18^

Histopathological evidence is the final confirmatory proof for the disease. The classical triad of dense lymphoplasmacytic infiltrates, storiform fibrosis, and obliterative phlebitis seen in the background of IgG4-positive plasma cells from the tumefactive lesions is the hallmark sign of IgG4-RD. Presence of all the three features makes the diagnosis distinct. Special stains such as elastin van Gieson (EVG) can be useful for visualising these features.^11^

### Diagnostic Criteria

Several diagnostic criteria have been used for establishing the diagnosis of IgG4-RD. The 2020 Japanese Revised Comprehensive Diagnostic (RCD) is an update of the 2011 criteria based on clinical/radiological, serological, and pathological features of the disease (**Table 3**).^19^ The diagnosis can be definite, probable, or possible based on the items satisfied in the criteria. The 2019 American College of Rheumatology/European League Against Rheumatism classification (ACR/EULAR) criteria involves a three-step diagnostic approach – inclusion step for potential cases, exclusion criteria step, and a step for scoring results of important criteria.^20^ The Mayo Clinic HISORt criteria is used for the diagnosis of AIP based on histopathology, imaging/serology, and response to steroid therapy.^21^

### Management

**Figure 3.**
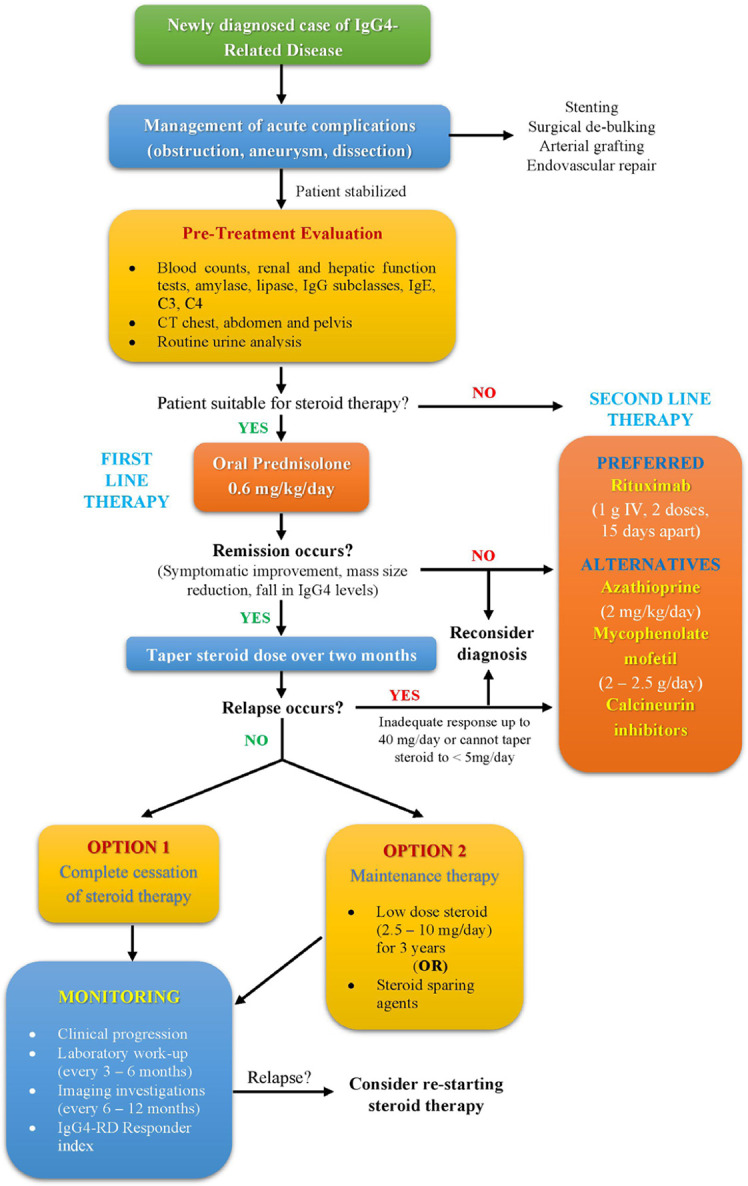
Management protocol for IgG4-related disease.

Early initiation of treatment is crucial in IgG4-RD in order to halt disease progression and prevent recurrence. Treatment outcomes depend on the degree of fibrosis, development of complications, and treatment adherence. Patients with IgG4-RD show good response to steroid therapy, however the duration of response is variable and flare-ups are common following dose tapering.^14^ The International Consensus Guidance Statement on the Management and Treatment of IgG4-RD suggests the initiation of treatment for all symptomatic, active cases as well as asymptomatic, progressive cases (**Figure 3**).^22^ A ‘wait-and-watch’ approach is advised for asymptomatic, non-progressive, limited disease with careful monitoring every six months.

A comprehensive pre-treatment evaluation is warranted prior to starting the treatment. Baseline complete cell counts, renal function tests, serum amylase/lipase, serum levels of IgG subclasses, IgE, and complements C3 and C4, and urine analysis must be done before treatment. Computed tomography of chest, abdomen and pelvis should be considered for identifying all foci of baseline disease activity.

Initial therapy of choice to induce remission is oral prednisolone at a dose of 0.6 mg/kg/day.^22,23^ Patients usually display a good response in 2 – 4 weeks. Once remission is achieved, the dose is tapered over a two-month period. The expert opinion is in favour of complete cessation of steroid therapy within three years in view of its long-term complications. A favourable outcome is seen in the form of symptomatic relief, reduction in the size of tumefactive mass or enlarged organ, and decrease in the serum IgG4 levels. In patients with multi-organ involvement or high baseline IgG4 levels, an induction therapy of glucocorticoids and an immunosuppressive agent such as rituximab may be considered.^22^

In cases of steroid dependence (inability to reduce steroid requirement below 5 mg/day), steroid resistance (inadequate response to steroids up to 40 mg/day), or cases with contraindications for steroids, two doses of the anti-CD20 antibody, rituximab (1 g intravenous) can be given two weeks apart.^22^ Azathioprine (2 mg/kg/day) and Mycophenolate mofetil (up to 2.5 g/day) can be considered as potential alternatives to rituximab.^22^ In cases of resistance to rituximab, switching over to the alternative immunosuppressant drugs or monoclonal antibodies such as abatacept (CD80/86 inhibitor) and dupilumab (IL-4 receptor blocker) have been proven to be helpful.^24,25^ It is however prudent to reconsider the diagnosis of IgG4-RD in cases of steroid resistance as the ACR/EULAR criteria considers ‘failure to respond to an appropriate glucocorticoid dose’ as an exclusion criterion for the disease.^20^

Administration of a maintenance therapy after a successful course of induction therapy is a matter of debate. Some physicians prefer to keep their patients under low-dose glucocorticoids (2.5 – 10 mg/day for three years) or a steroid-sparing agent as a means of reducing the risk of relapse. Recurrent disease is a clinical dilemma of concern – retreatment with steroids is considered if relapse occurs after successful remission, however relapses during the course of steroid therapy requires the use of steroid-sparing agents such as rituximab, azathioprine, mycophenolate mofetil, and calcineurin inhibitors.^22^

Active research is underway to bring forth newer therapeutic drugs for IgG4-RD. As of 2025, inebilizumab (UPLIZNA^®^, humanised anti-CD19 cytolytic monoclonal antibody) is the only FDA-approved drug for the disease. The results of the Phase 3 MITIGATE trial show a reduction in the disease flare risk up to 87% with inebilizumab as compared to the control group.^26^ It is contraindicated in patients with fatal infusion reactions, active hepatitis B, or untreated tuberculosis. Other biologics such as belimumab, obexelimab, rilzabrutinib, and zanubrutinib are yet in the pipeline for their application in IgG4-RD.^27^

Surgical intervention may play a limited role in the disease management. Obstructive pathologies such as hydronephrosis (in retroperitoneal fibrosis) and obstructive jaundice (in biliary disease) require stenting procedures to relieve the acute retention and facilitate drainage. Tumefactive mass lesions may require surgical de-bulking to prevent organ damage and vascular compromise. Cases of aortitis and aneurysms are managed with arterial graft replacement and endovascular repair. It is worth noting that the success of surgical interventions in IgG4-RD is dependent on the concomitant administration of medical therapy.^14,22^

Regular monitoring is required for all patients of IgG4-RD to assess the disease activity, progression and treatment response. Patients should be advised to be wary of new symptoms that may arise along the treatment course, and could be a sign of newly active disease at a different site. Evaluation of cell counts, blood chemistries, IgG subclasses, IgE, and C3/C4 levels must be done every 3 – 6 months. Imaging studies should be done every 6 – 12 months to assess disease progression.^22^ IgG4-RD Responder Index (IgG4-RD RI) is a useful tool to assess disease burden and treatment response in patients with IgG4-RD.^28^

## DISCUSSION

The purpose of the case discussion and review is to illustrate the diversity in the clinical presentation of IgG4-RD, and illustrate how seemingly unrelated findings can point to the diagnosis of IgG4-RD with astute clinical observation and a prior knowledge of the disease presentations. We wish to educate clinicians about the disease and encourage them to consider IgG4-RD as a possible differential diagnosis in their patients. Several factors contribute to the uniqueness of our case discussion. Our patient is a rare confluence of two different subsets of the IgG4-RD spectrum, namely pancreatic involvement and head and neck disease. The prevalence of orbital manifestations (orbital pseudotumor, in our case) in patients with pancreato-hepatobiliary disease is less than 1%.^16^ The occurrence of autoimmune pancreatitis alongside orbital pseudotumor without salivary/lacrimal gland involvement in a female patient (in contrary to the male predilection) is an unusual presentation of the disease captured in our work. Through this work, we wish to highlight on the heterogeneity in clinical presentation of the disease, and emphasise on the need for clinicians to screen all potential foci of disease, despite predominance of any specific disease sub-group in the patients.

This work is the first documentation of the practice of using higher initial steroid dosage (1 mg/kg/day) for reducing the risk of disease relapse among the Indian practitioners. While no prior published study robustly demonstrates this regional variation, evidence is accumulating at our institution to support this practice. Venous thrombosis is quite a rare complication of IgG4-RD. Previous literature has cases of deep vein/inferior vena cava thrombosis and cerebral venous thrombosis associated with IgG4-RD.^29,30^ It is often seen in conjunction with retroperitoneal fibrosis, owing to the mechanical compression imparted on the retroperitoneal vessels, thus altering the flow dynamics. Apart from this, the experts believe that the chronic inflammatory state in the disease must also mediate endothelial dysfunction and confer a pro-thrombotic state, thus predisposing venous thrombosis.^31^ The findings in our patient have revealed a partial inferior vena cava and segmental renal vein thrombosis in the absence of any retroperitoneal disease or thrombophilic condition, adding to the rarity of case. Our experience of successfully handling this thrombotic presentation with intravenous heparin and oral apixaban could guide practitioners experiencing a similar dilemma in the future.

A strong clinical suspicion is required for the diagnosis of IgG4-related disease. Subacute-to-chronic duration of constitutional symptoms, tumefactive lesions, and multi-organ manifestations in a middle-aged/elderly individual are clues pointing towards the disease. IgG4-RD must be considered as a differential diagnosis for mass lesions and lymphadenopathy alongside malignancies and infections. Autoimmune disease screening protocols need to include IgG4-RD due to the significant overlap between the conditions.

## CONCLUSION

The purpose of this review is to demonstrate that considerable variability is to be expected in the clinical presentations of the disease, as it may involve virtually any organ in the body. However, it is interesting to note that one will find elevated serum IgG4 levels and strikingly similar histopathological findings (dense lymphoplasmacytic infiltrates, storiform fibrosis and obliterative phlebitis) in almost all forms of disease involvement. Thus, it is prudent to evaluate these parameters in case of suspicion for the disease.

Once identified, a thorough pre-treatment evaluation and prompt initiation of steroid therapy can check the disease progression and prevent complications. A dramatic response to steroids is a hallmark sign and can be considered retrospective evidence for the disease. Regular monitoring of disease activity is essential to assess treatment response, identify new foci of disease, and tailor therapeutic regimens for the patients.
